# Effects of TGF-*β*1 on OPG/RANKL Expression of Cementoblasts and Osteoblasts Are Similar without Stress but Different with Mechanical Compressive Stress

**DOI:** 10.1155/2015/718180

**Published:** 2015-01-15

**Authors:** Xianrui Yang, Yanmin Wang, Xianglong Han, Rui Shu, Tian Chen, Huan Zeng, Xin Xu, Lan Huang, Aishu Ren, Jinlin Song, Li Cao, Ding Bai

**Affiliations:** ^1^State Key Laboratory of Oral Diseases, Department of Orthodontics, West China Hospital of Stomatology, Sichuan University, Chengdu 610041, China; ^2^Chongqing Key Laboratory for Oral Diseases and Biomedical Sciences, Department of Orthodontics, The Affiliated Hospital of Stomatology, Chongqing Medical University, Chongqing 400016, China

## Abstract

*Introduction.* This study aimed to explore the effects of TGF-*β*1 on regulating activities of cementoblasts and osteoblasts with or without stress.* Material and Methods*. Human recombinant TGF-*β*1 was added with different doses. Immunohistochemical test of osteoprotegerin (OPG)/receptor activator of nuclear factor-kappaB ligand (RANKL) and Alizarin Red-S staining were conducted. Mechanical compressive stress was obtained by increasing the pressure of gaseous phase. OPG/RANKL expression was detected in both cells through quantitative real-time PCR. *Results*. Similar significant differences (*P* < 0.05) existed in OPG/RANKL change with increasing concentration of TGF-*β*1 without mechanical stress for cementoblasts and osteoblasts. However, under 3 h stress, OPG increased and RANKL decreased significantly (*P* < 0.01) but with similar OPG/RANKL change. Moreover, under 24 h stress, OPG change exhibited no difference (*P* > 0.05), but RANKL decreased significantly (*P* < 0.01) at 10 and 100 ng/mL TGF-*β*1 in cementoblasts. In osteoblasts, OPG increased significantly (*P* < 0.01) at 10 and 100 ng/mL, whereas RANKL decreased with statistical difference (*P* < 0.05) at 1 and 10 ng/mL. *Conclusions*. The effects of TGF-*β*1 on OPG/RANKL expression of cementoblasts and osteoblasts are similar even without mechanical stress. However, these effects are different under mechanical compressive stress.

## 1. Introduction

Cementum is a special mineralized tissue covering the root surface of teeth; this tissue assists in anchoring teeth to alveolar bone and contributes to the maintenance of dentition, structural stability, and physiological function of teeth. Although teeth share similar properties and biochemical composition with those of bones, the teeth differ from bones in histological profile by lacking innervation and vascularization with limited remodeling potential [1]. Cementoblasts, which mainly comprise cementum, also share many similar properties with those of osteoblasts, which are the essential components of bones. As force-sensitive type of cells, both cementoblasts and osteoblasts change their functions and activities under mechanical stress to regulate the resorption and formation of bone and cementum [2]. Although some studies showed that cementoblasts may differ from osteoblasts regarding responses to mechanical stresses [3], this assumption remains controversial. The osteogenic cell lineage MC3T3-E1, which behaves similarly to primary osteoblasts, and the cementogenic cell lineage OCCM-30, which expresses specific cementum-derived attachment protein and differentiates into terminally differentiated cementocytes, were considered good models for in vitro studies [4, 5].

The osteoprotegerin (OPG)/receptor activator of nuclear factor-kappaB ligand (RANKL) system is essential during bone metabolism [6]. OPG belongs to the tumor necrosis factor receptor superfamily and exhibits vital protective function for bones. OPG functions as a decoy receptor by binding to RANKL. Therefore, RANK and RANKL interaction was prevented, and both development and activity of osteoclasts were inhibited [7]. In cementoblasts, similar expression of OPG and RANKL has been detected in periodontal ligament cells in vitro [8]. The OPG/RANKL system participates in modulation of osteoblast-mediated osteoclastogenesis influencing alveolar remodeling, as well as in root cementum resorption. Although bone remodeling is related to the OPG/RANKL system, protective mechanisms are necessary to prevent root cementum resorption [9].

Transforming growth factor-*β*1 (TGF-*β*1) plays a major role in the development and maintenance of skeletal tissues, thereby affecting bone metabolism [10]. As a ubiquitous growth factor, TGF-*β*1 regulates cell proliferation, migration, differentiation, and survival; TGF-*β*1 also functions in diverse processes, such as embryogenesis and wound healing. Previous studies reported that osteoblasts can produce TGF-*β*1, which is one of the most important factors in the bone milieu, thereby retaining the balance between the dynamic processes of bone resorption and formation [11]; TGF-*β*1 also influences the OPG/RANKL system in osteoblasts [12]. Notably, the cementoblastic response to TGF-*β*1 should be characterized to influence the OPG/RANKL expression. However, the contribution of TGF-*β*1-treated cementoblasts to the production of OPG and RANKL remains unknown. This study was designed to evaluate the effects of TGF-*β*1 on the regulation of cementoblast-mediated osteoclastogenesis by using a well-established cementoblastic cell line (OCCM-30) and an osteoblastic cell line (MC3T3-E1) with or without mechanical compressive stress.

## 2. Material and Methods

### 2.1. Cell Line and Cell Culture

An immortalized murine cementoblast cell line (OCCM-30, kindly provided by Professor Somerman, Washington, USA) and a murine osteoblast cell line (MC3T3-E1) were used. OCCM-30 was maintained in Dulbecco's Modified Eagle's Medium/Nutrient Mixture F12 (DMEM/F12; Gibco, USA) with 10% fetal bovine serum (FBS; Chengdu, China), 100 U/mL penicillin, and 100 *μ*g/mL streptomycin in a 37°C, humidified, 5% CO_2_/95% air environment. Once the cells proliferated and reached 80% confluence, DMEM/F12 without FBS was used for cell-cycle synchronization. The medium was changed every 2 d.

### 2.2. TGF-*β*1 Interference

Cells were incubated at 37°C in a humidified atmosphere of 95% air and 5% CO_2_. When cells reached subconfluence, they were detached accordingly and seeded onto prepared coverslips in six-well plates at 2 × 10^5^ cells/mL concentration with a total 2 mL for each plate. DMEM/F12 without FBS was used for cell-cycle synchronization for 24 h incubation. Subsequently, the cells were seeded into the plates for 48 h. Human recombinant TGF-*β*1 (Protech Technology, Sparks, NV, USA) of different concentrations at 1, 10, and 100 ng/mL, treated with 0.1% FCS and negative control (DMEM/0.1% FCS of equal quantity), was added to OCCM-30 and MC3T3-E1, respectively, at 48 h before mechanical stimulation.

### 2.3. Immunohistochemistry

TGF-*β*1-treated (1, 10, and 100 ng/mL) OCCM-30 and MC3T3-E1 were washed, fixed in 10% formalin, and blocked in 5% normal serum. Cells were probed with primary antibody pSMAD1/5/8 (1 : 100; Cell Signaling) overnight at 4°C and visualized with secondary antibody conjugated to Alexa Fluor 488 (1 : 200; Invitrogen) for 30 min. ProLong Gold antifade reagent with 4,6-diamidino-2-phenylindole (Invitrogen P36935) was used for mounting. The control group used phosphate-buffered saline (PBS) instead of primary antibody, but all the other steps were performed the same as those for the experimental groups.

### 2.4. Alizarin Red-S Staining

OCCM-30 and MC3T3-E1 after TGF-*β*1 treatment (1, 10, and 100 ng/mL) were washed thrice with PBS. Cells were fixed with 95% ethanol for 30 min and allowed to dry completely. Alizarin Red-S solution (0.1% Alizarin Red Tris-HCl, pH 4.3) was added to the plates, which were incubated for 30 min at room temperature (37°C). The cells were carefully rinsed thrice with double-distilled water and then allowed to dry. Stained cells were examined and photographed under a light microscope.

### 2.5. Mechanical Compressive Stress Condition

Mechanical stimulation was achieved by increasing the pressure of the gaseous phase above the media in this study. The pressure machine adopted was custom-made and computer-operated by the national state laboratory, and the details of the machine were reported previously [13]. The machine was used to mimic the force that teeth underwent. Static pressure surroundings were maintained inside a sealed chamber (37°C, 5% CO_2_/95% air, humidified environment) where the cells were mechanically stimulated. When OCCM-30 and MC3T3-E1 reached subconfluence, they were detached accordingly and seeded onto prepared coverslips in six-well plates at 2 × 10^5^ cells/mL concentration with a total of 2 mL in each plate for 48 h. DMEM/F12 without FBS was used for cell-cycle synchronization for another 24 h. OCCM-30 and MC3T3-E1 were then exposed to 23 kPa static pressure for 3 and 24 h, respectively. Control cells were cultured in the same way without loading pressure.

### 2.6. Quantitative Real-Time Polymerase Chain Reaction

OCCM-30 and MC3T3-E1 after treatment (TGF-*β*1 and mechanical stimulation) were washed with PBS. Total cellular RNA was extracted using Trizol reagent (Invitrogen, USA) according to the manufacturer's instructions. DNase-treated RNA was reverse-transcribed to synthesize cDNA using the Takara RT-PCR kit (Takara, Japan). Real-time PCR was then performed in an ABI PRISM 7300 Real-Time PCR System. Primer sequences, which were self-designed with Primer Premier 5.0 software (Premier Biosoft International, CA, USA) for each gene encoding, are as follows: OPG (5′-TCAGAAAGGAAATGCAACACA-3′/5′-CCGTTTTATCCTCTCTACACT-3′); RANKL (5′-CCGTTTTATCCTCTCTACACT-3′/5′-TTAGGATCCATCTGCGCTC-3′); and GAPDH (5′-CCTCAAGATTGTCAGCAAT-3′/5′-CCATCCACAGTCTTCTGGGT-3′). The housekeeping gene GAPDH was concurrently amplified in each sample as a reference gene and was used for normalization.

### 2.7. Statistical Analysis

All experiments were performed four times with comparable results. Data are expressed as mean ± SD for each group if no statistical difference existed in the variations among the four instances. Significance was assessed by ANOVA using SPSS software package (version 18.0, SPSS, Chicago, IL). The level of significance was set at 0.05.

## 3. Results

### 3.1. Identification of Cells

**Figure 1:**
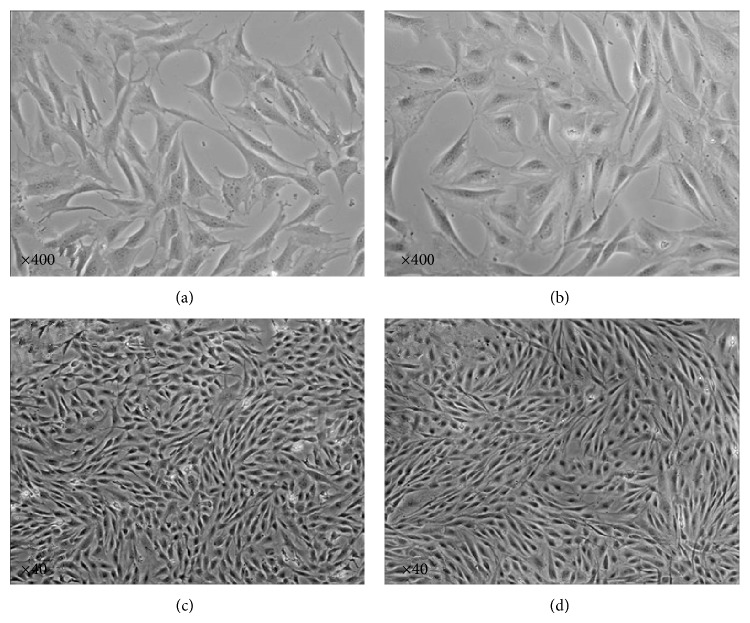
Morphological observation of cementoblasts and osteoblasts under microscope.

Cell viability of OCCM-30 and MC3T3-E1 was analyzed using Vi-CELLTM cell viability analyzers (Beckman Coulter Inc., USA) after 24 h culture without FBS and observed under an inverted phase contrast microscope (Figure 1).

Figure 1 shows the morphology of cementoblasts (OCCM-30) with inverted phase contrast microscope in different magnifications at ×400 (a) and ×40 (c) and osteoblasts (MC3T3-E1) in magnifications at ×400 (b) and ×40 (d).

### 3.2. Immunohistochemical Results of OPG and RANKL

**Figure 2:**
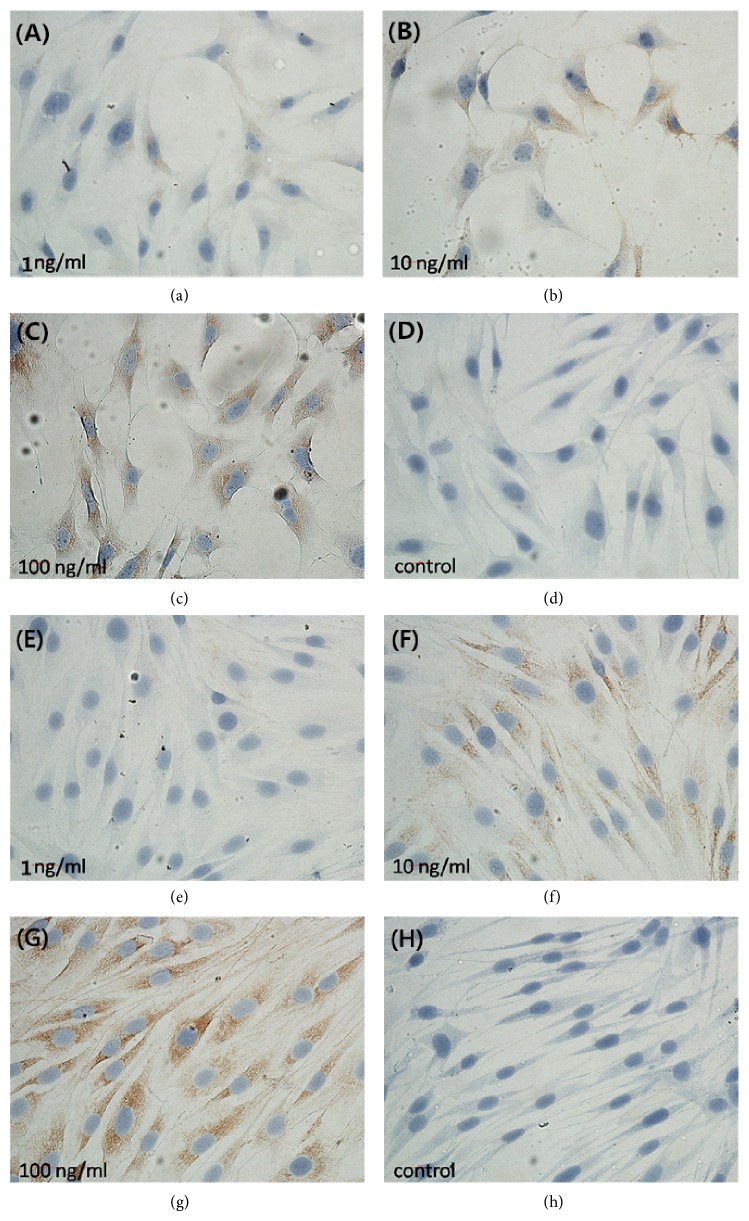
Immunohistochemical results of OPG expression in OCCM-30 and MC3T3-E1 cells with TGF-*β*1 treatment.

**Figure 3:**
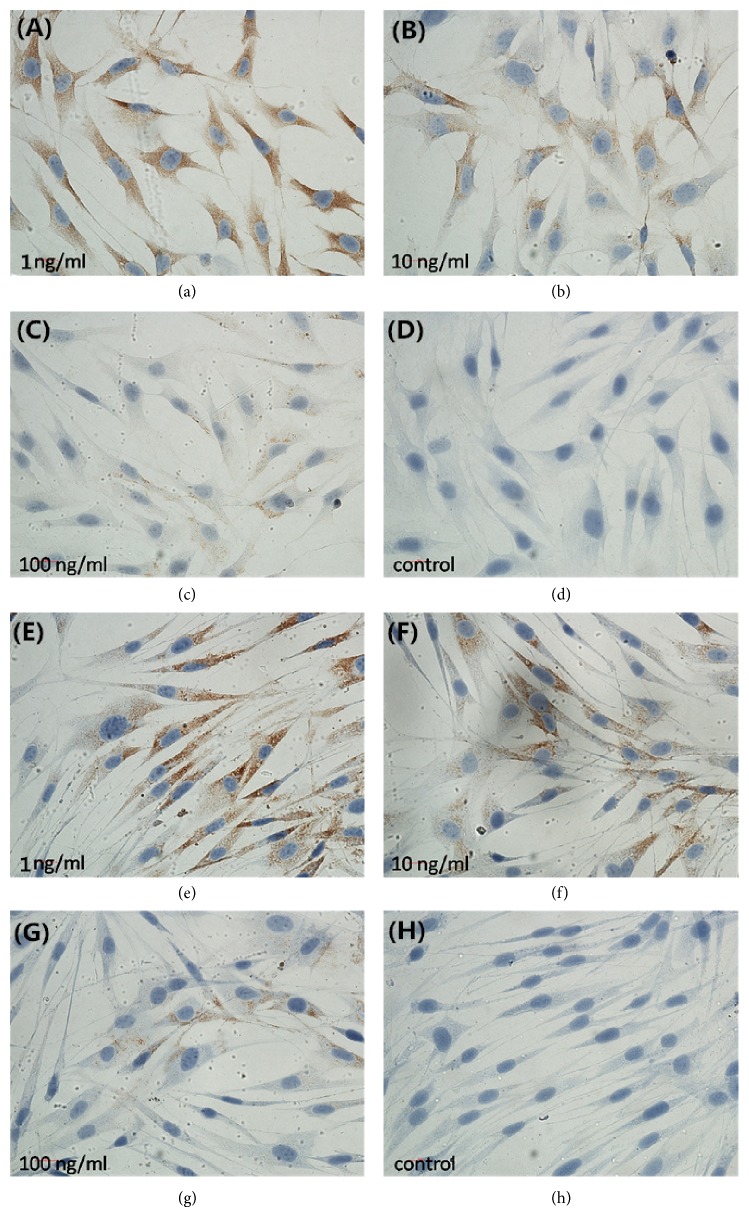
Immunohistochemical results of RANKL expression in OCCM-30 and MC3T3-E1 cells with TGF-*β*1 treatment.

With the increasing TGF-*β*1 concentrations at 1, 10, and 100 ng/mL, OPG expression also increased in OCCM-30 and MC3T3-E1 cells (Figure 2), whereas RANKL expression decreased in both cells (Figure 3).

Figure 2 shows immunohistochemical results of OPG expression in OCCM-30 cells with TGF-*β*1 concentrations at 1 (a), 10 (b), and 100 ng/mL (c) compared with control group (d), as well as in MC3T3-E1 cells with TGF-*β*1 concentrations at 1 (e), 10 (f), and 100 ng/mL (g) compared with control group (h). With increasing TGF-*β*1 concentration, the brown area also increased as indicated by increasing OPG expression.

Figure 3 shows the immunohistochemical results of RANKL expression in OCCM-30 cells with TGF-*β*1 concentrations at 1 (a), 10 (b), and 100 ng/mL (c) compared with control group (d), as well as in MC3T3-E1 cells with TGF-*β*1 concentrations at 1 (e), 10 (f), and 100 ng/mL (g) compared with control group (h). With increasing TGF-*β*1 concentration, the brown area decreased as indicated by decreasing RANKL expression.

### 3.3. Alizarin Red-S Staining of Mineralization

**Figure 4:**
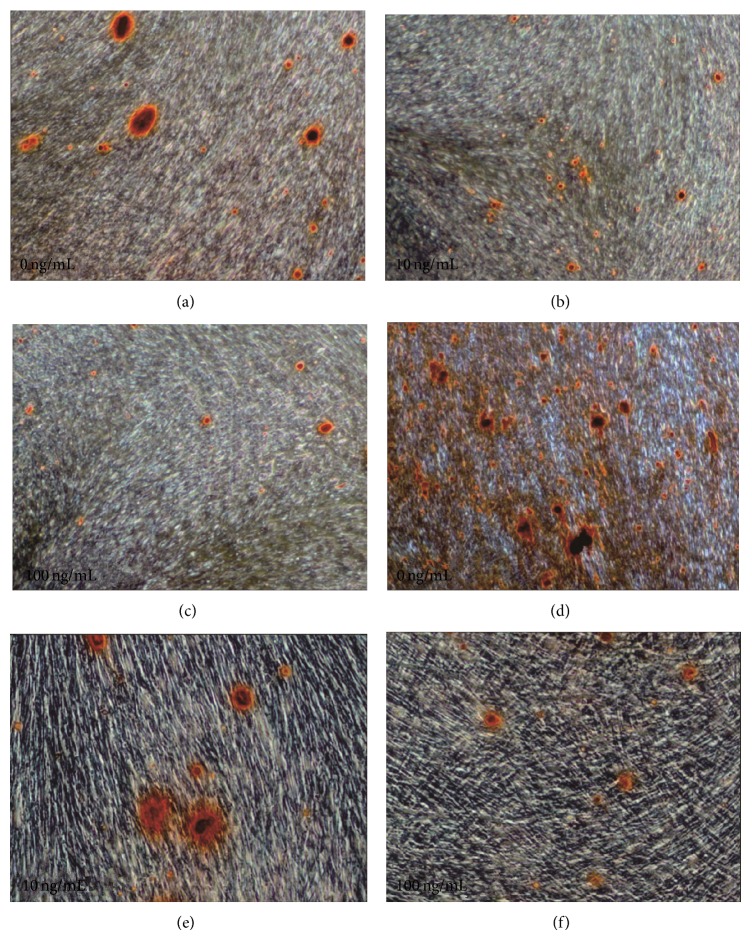
Alizarin Red-S staining results of mineralization in OCCM-30 and MC3T3-E1 cells with TGF-*β*1 treatment.

As the TGF-*β*1 concentration increased from 1 ng/mL to 10 and 100 ng/mL, the mineralization of OCCM-30 and MC3T3-E1 cells decreased accordingly. This finding indicated that TGF-*β*1 exhibits a negative effect on mineralization in cells, and the influence is more significant in OCCM-30 than in MC3T3-E1 (Figure 4).

Figure 4 shows the results of Alizarin Red-S staining of OCCM-30 cells with TGF-*β*1 concentrations at 1 (a), 10 (b), and 100 ng/mL (c), as well as with MC3T3-E1 at 1 (d), 10 (e), and 100 ng/mL (f). The brown area indicates that mineralization weakened as TGF-*β*1 concentration increased.

### 3.4. Effects of TGF-*β*1 without Mechanical Stress

**Figure 5:**
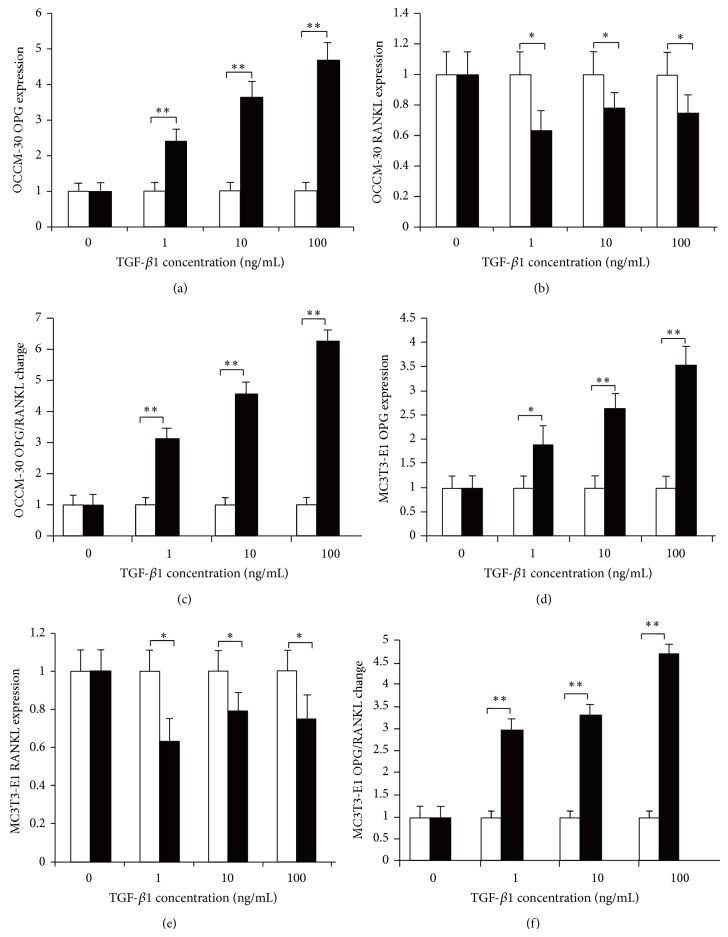
Effects of TGF-*β*1 on cementoblasts and osteoblasts without mechanical stress.

Quantitative real-time PCR results of TGF-*β*1 concentration change without mechanical stress indicated that, with increasing TGF-*β*1 concentration, OPG expression in both OCCM-30 and MC3T3-E1 cells was increased correspondingly with statistical significance (*P* < 0.05). However, the RANKL expression decreased without correspondence but with statistical difference (*P* < 0.05) and was similar between OCCM-30 and MC3T3-E1, as well as in the increasing OPG/RANKL ratio (Figure 5).

Figure 5 shows the expression of OPG and RANKL in OCCM-30 and MC3T3-E1 without mechanical stress. With increasing TGF-*β*1, OPG expression in OCCM-30 increased significantly (a), whereas RANKL expression in OCCM-30 decreased significantly (b), thereby increasing OPG/RANKL correspondingly (c). Similarly, in MC3T3-E1, OPG expression increased (d), whereas RANKL decreased (e) with statistical difference, and OPG/RANKL increased (f): ^*^ *P* < 0.05 and ^**^ *P* < 0.01.

### 3.5. Effects of TGF-*β*1 under 3 h Mechanical Compressive Stress

**Figure 6:**
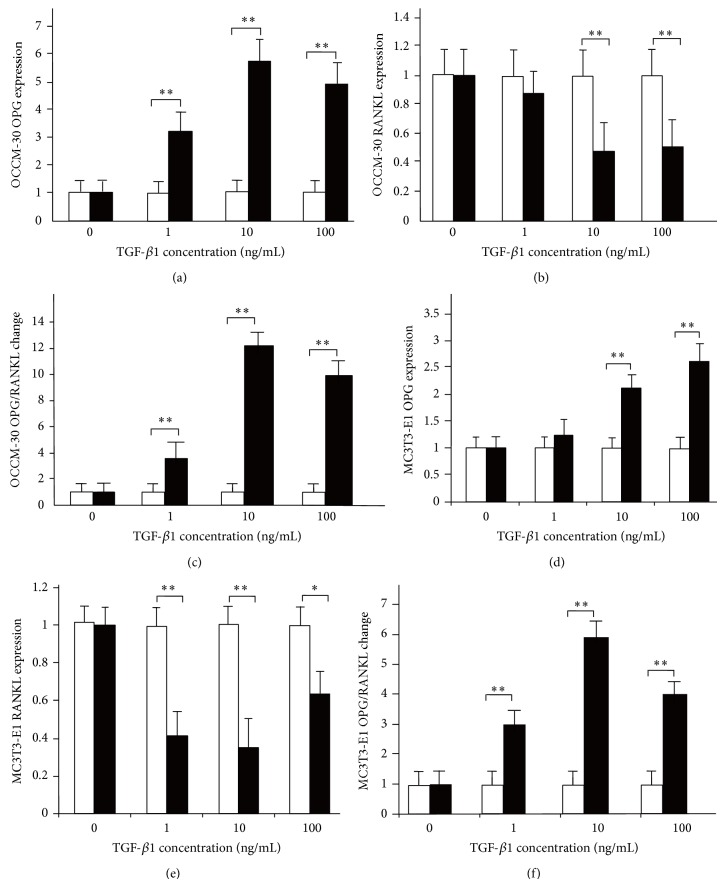
Effects of TGF-*β*1 on cementoblasts and osteoblasts under 3 h mechanical compressive stress.

When the cells underwent 3 h mechanical compressive stress with different TGF-*β*1 concentrations, the OPG expression increased significantly at the TGF-*β*1 concentrations of 1 (*P* < 0.01), 10 (*P* < 0.01), and 100 ng/mL (*P* < 0.01) in OCCM-30 cells. The most significant increase occurred at 10 ng/mL concentration. In MC3T3-E1 cells, the increase corresponded and differed significantly at concentrations of 10 (*P* < 0.01) and 100 ng/mL (*P* < 0.01). In the RANKL expression, both cells decreased with the increased concentration of TGF-*β*1. In OCCM-30 cells, the decrease differed significantly (*P* < 0.01) at concentrations of 10 and 100 ng/mL, whereas, in MC3T3-E1 cells, the decrease differed statistically at the concentration of 100 ng/mL (*P* < 0.05) and significantly at the concentrations of 1 (*P* < 0.01) and 10 ng/mL (*P* < 0.01). The OPG/RANKL ratio change trend was also similar but more significant in OCCM-30 than in MC3T3-E1 (Figure 6).

Figure 6 shows the expression of OPG and RANKL in OCCM-30 and MC3T3-E1 under 3 h mechanical compressive stress. With increased TGF-*β*1, OPG expression in OCCM-30 increased significantly, particularly at concentration of 10 ng/mL (a), whereas RANKL expression in OCCM-30 decreased significantly at concentrations of 10 and 100 ng/mL (b); OPG/RANKL ratio increased mostly at 10 ng/mL (c). In MC3T3-E1, OPG expression increased significantly at 10 and 100 ng/mL (d), whereas RANKL expression decreased (e) with statistical difference, particularly at concentration of 10 ng/mL, and OPG/RANKL ratio also changed (f): ^*^ *P* < 0.05 and ^**^ *P* < 0.01.

### 3.6. Effects of TGF-*β*1 under 24 h Mechanical Compressive Stress

**Figure 7:**
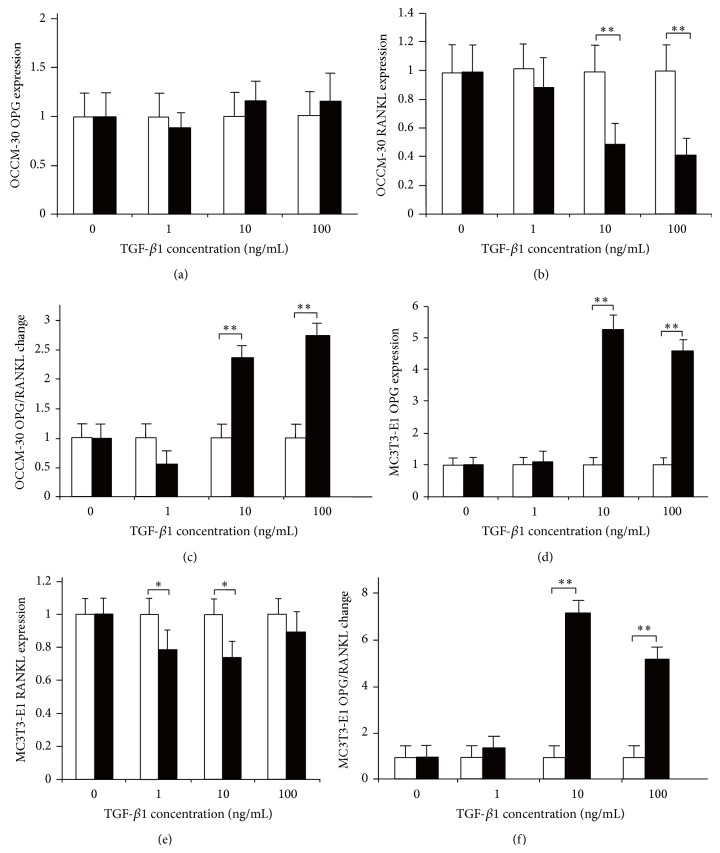
Effects of TGF-*β*1 on cementoblasts and osteoblasts under 24 h mechanical compressive stress.

The results of 24 h mechanical compressive stress in OCCM-30 and MC3T3-E1 cells differed with increased TGF-*β*1 concentration. In OCCM-30 cells, the OPG expression changed without statistical significance (*P* > 0.05), and the RANKL expression decreased significantly at concentrations of 10 and 100 ng/mL. However, in the MC3T3-E1 cells, the OPG expression slightly increased without statistical difference at 1 ng/mL concentration (*P* > 0.05) but significantly at 10 (*P* < 0.01) and 100 ng/mL (*P* < 0.01). The RANKL expression decreased with statistical difference at the concentrations of 1 (*P* < 0.05) and 10 ng/mL (*P* < 0.05) but differed without significance at 100 ng/mL (*P* > 0.05). The OPG/RANKL ratio also increased more in OCCM-30 than in MC3T3-E1 (Figure 7).

Figure 7 shows the expression of OPG and RANKL in OCCM-30 and MC3T3-E1 under 24 h mechanical compressive stress. With increased TGF-*β*1, OPG expression in OCCM-30 was insignificant (a), whereas RANKL expression in OCCM-30 decreased significantly at concentrations of 10 and 100 ng/mL (b). OPG/RANKL ratio also decreased at 1 ng/mL but increased at 10 and 100 ng/mL (c). In MC3T3-E1, OPG expression increased significantly at concentrations of 10 and 100 ng/mL (d), whereas RANKL decreased with statistical difference at 1 and 10 ng/mL (e), and OPG/RANKL ratio changed (f): ^*^ *P* < 0.05 and ^**^ *P* < 0.01.

### 3.7. Tendency of OPG and RANKL Expression

**Figure 8:**
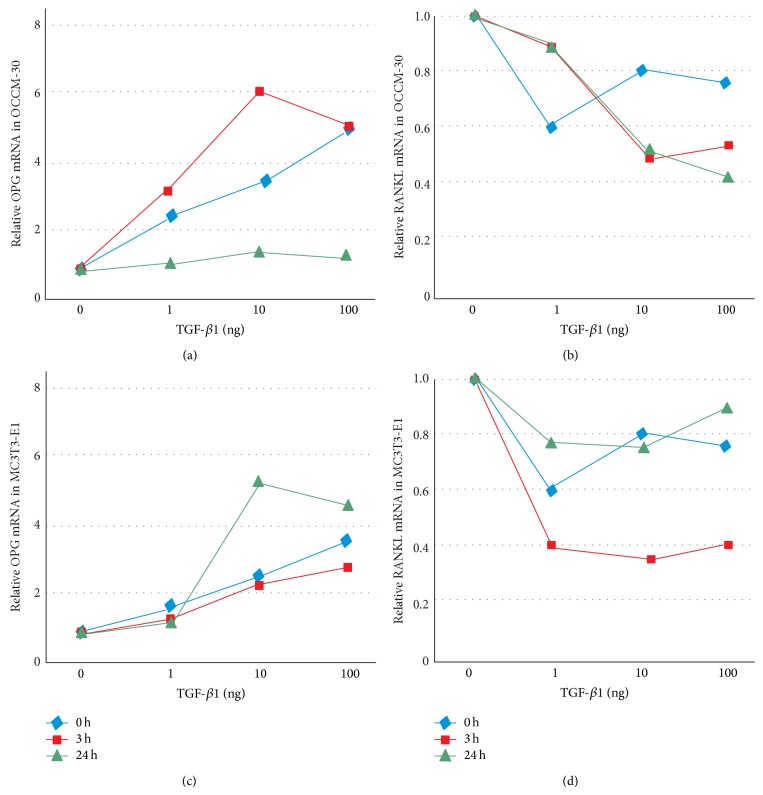
Change tendencies of OPG and RANKL under different mechanical compressive stress durations.

The expression change of OPG and RANKL exhibited similar tendency in OCCM-30 and MC3T3-E1 cells when the duration of mechanical compressive stress changed from 0 h and 3 h to 24 h. However, the change process under different stress durations and TGF-*β*1 concentrations varied (Figure 8).

Under mechanical compressive stress in different durations of 0, 3, and 24 h, as well as change tendency of OPG expression (a) and RANKL expression (b) in OCCM-30 cells, increasing OPG expression and decreasing RANKL differ with various stress durations. Figure 8 also shows change tendencies in OPG expression (c) and RANKL expression (d) in MC3T3-E1 cells, as indicated by difference with OCCM-30 cells.

## 4. Discussion

Although cementoblasts and osteoblasts share many similar properties, they still differ in some characteristics. Osteoclastogenesis and bone resorption are mainly regulated by OPG and RANKL [14]. With RANKL binding to RANK on preosteoclasts, osteoclastogenesis is initiated. OPG, a secreted glycoprotein, acts as a decoy receptor by binding to RANKL and inhibits osteoclastogenesis [15]. OPG and RANKL can be modulated by various factors [16], such as TGF-*β*1. TGF-*β*1 affects osteoblast differentiation, matrix formation, and mineralization but negatively regulates osteoclastogenesis by increasing levels of OPG and decreasing RANKL in osteoblasts [17] and cementoblasts [18]. Given that TGF-*β*1 plays a critical role in regulating both cementoblasts and osteoblasts, the effects on these cells may differ. However, this study showed that these effects were similar in the condition without mechanical stress; TGF-*β*1 exposure induced upregulation of OPG and downregulation of RANKL. Thus, OPG/RANKL was increased, which may explain the importance of TGF-*β*1 in protecting the bone and cementum of the root surface from resorption. Many previous studies reported that TGF-*β*1 can promote wound healing and periodontal tissue regeneration [19]. TGF-*β*1 also exhibits a complex influence on OPG/RANKL in osteoblasts. RANKL expression can increase with low concentration of TGF-*β*1; by contrast, with increasing TGF-*β*1 concentration, RANKL expression will decrease and OPG expression will increase [20]. These findings correspond with our current results. However, almost no previous research has investigated the influence of TGF-*β*1 on cementoblasts compared with osteoblasts, which we reported in the present study. As for the mechanical loading, a study [21] also found that osteoblasts and cementoblasts exhibit distinct responses despite similar biochemical markers expressed; differential genetic responses may cause such difference, which we also reported in the current research.

Under mechanical compressive stress, the TGF-*β*1 effect on cementoblasts and osteoblasts differed in 3 and 24 h duration. The present study used a gaseous filled unit to load a mechanical stress of 23 KPa as previously reported [22, 23]. When the stress sustained 3 h, cementoblasts expressed more OPG when 1 ng/mL TGF-*β*1 was added, whereas osteoblasts expressed more OPG when 10 ng/mL TGF-*β*1 was added. These findings indicated that OPG expression in cementoblasts may be much sensitive to TGF-*β*1 under 3 h mechanical compressive stress. Oppositely, for RANKL expression, cementoblasts expressed less RANKL when 10 ng/mL TGF-*β*1 was added, whereas osteoblasts expressed less RANKL when 1 ng/mL TGF-*β*1 was added. The OPG/RANKL ratio change trend was similar between both cells but much higher in cementoblasts than in osteoblasts. This result indicated that TGF-*β*1 may exhibit more effects on cementoblasts under 3 h mechanical compressive stress. When the stress sustained 24 h, OPG expression in cementoblasts changed with very little irregularity as the TGF-*β*1 concentration increased. By contrast, OPG expression increased significantly in osteoblasts at concentrations of 10 and 100 ng/mL, which implied that OPG expression in cementoblasts is inert to TGF-*β*1, whereas osteoblasts are active at certain concentration of TGF-*β*1 under 24 h mechanical compressive stress. As for RANKL decrease, cementoblasts changed at the concentrations of 10 and 100 ng/mL, whereas osteoblasts changed at 1 and 10 ng/mL. These findings indicated that the RANKL expression of cementoblasts and osteoblasts reacted to TGF-*β*1 at different points. The OPG/RANKL ratio change was also higher in osteoblasts than in cementoblasts. This result predicted that TGF-*β*1 may exhibit more effects on osteoblasts under 24 h mechanical compressive stress. Therefore, we should consider not only the influence of mechanical stress duration but also the different reactions of cementoblasts or osteoblasts when using TGF-*β*1 to regulate both cells.

TGF-*β*1 plays a critical role in bone remodeling [24], and the mechanism may be related to ERK and JNK signal pathways or through MAPK pathway in regulating Smad signal. TGF-*β*1 combined with BMPs can also induce Runx2 expression. This expression may activate Smad3 and interact with Runx2 to inhibit gene expression of other osteoblasts, such as collagen I, ALP, and osteocalcin, via self-regulation feedback mechanism. Overexpression of Smad2 would also decrease the expression of Runx2 mRNA. Both osteocytes and cementocytes can express sclerostin, which is a Wnt signaling antagonist that controls bone remodeling; the lack of sclerostin can alter bone and cementum phenotypes [25], which may also participate in this mechanism. However, the different effects on osteoblasts and cementoblasts under mechanical stress remain unclear because the effects are similar in both cells without stress. Thus, further studies are necessary. Other signal pathways may be involved in the reaction, or differential genetic responses to mechanical loading may provide functional markers to distinguish the cementoblast and osteoblast phenotypes [21].

The interpretation of the differences may be related to different functionalities, considering that cementoblasts participate more in very slow cementum remodeling after maturation, whereas osteoblasts are involved more in continuous bone remodeling with or without additional mechanical stress. Clinically, these cells regularly receive mechanical stress from occlusal force or orthodontic force in oral environment. The periodontal ligament or root may be damaged under unexceptional forces, and whether any method prevents destruction or protects those tissues remains controversial [26]. Some growth factors may help address the problem. Given that the mechanical stress of tooth movement differently affects the alveolar bone and cellular cementum [27], orthodontists should determine the difference of osteoblasts and cementoblasts in response to mechanical stress. The addition of growth factors, such as TGF-*β*1, as an exogenous application during periodontal tissue repair in orthodontic treatment may be an alternative therapeutic approach to prevent periodontal damage.

## 5. Conclusions

The effects of TGF-*β*1 on cementoblasts and osteoblasts are similar even without mechanical stress or upon inducing OPG expression and inhibiting RANKL expression. However, these effects are different between cementoblasts and osteoblasts under mechanical compressive stress. The expression change trends of OPG/RANKL are similar under 3 h compressive stress but higher in cementoblasts, and the change point and amount are different with TGF-*β*1 concentration change between cementoblasts and osteoblasts. Under 24 h mechanical compressive stress, TGF-*β*1 also affects the expression of OPG and RANKL in cementoblasts and osteoblasts differently, and the OPG/RANKL ratio change is higher in osteoblasts.

## References

[1] Komaki M., Iwasaki K., Arzate H., Narayanan A. S., Izumi Y., Morita I. (2012). Cementum protein 1 (CEMP1) induces a cementoblastic phenotype and reduces osteoblastic differentiation in periodontal ligament cells. *Journal of Cellular Physiology*.

[2] Kitagawa M., Tahara H., Kitagawa S. (2006). Characterization of established cementoblast-like cell lines from human cementum-lining cells in vitro and in vivo. *Bone*.

[3] D'Errico J. A., Macneil R. L., Takata T., Berry J., Strayhorn C., Somerman M. J. (1997). Expression of bone associated markers by tooth root lining cells, in situ and in vitro. *Bone*.

[4] Wang D., Christensen K., Chawla K., Xiao G., Krebsbach P. H., Franceschi R. T. (1999). Isolation and characterization of MC3T3-E1 preosteoblast subclones with distinct in vitro and in vivo differentiation/mineralization potential. *Journal of Bone and Mineral Research*.

[5] Mada Y., Miyauchi M., Oka H. (2006). Effects of endogenous and exogenous prostaglandin E2 on the proliferation and differentiation of a mouse cementoblast cell line (OCCM-30). *Journal of Periodontology*.

[6] Grcevic D., Katavic V., Lukic I. K., Kovacic N., Lorenzo J. A., Marusic A. (2001). Cellular and molecular interactions between immune system and bone. *Croatian Medical Journal*.

[7] Hofbauer L. C., Heufelder A. E. (2001). Role of receptor activator of nuclear factor-*κ*B ligand and osteoprotegerin in bone cell biology. *Journal of Molecular Medicine*.

[8] Hasegawa T., Yoshimura Y., Kikuiri T. (2002). Expression of receptor activator of NF-kappa B ligand and osteoprotegerin in culture of human periodontal ligament cells. *Journal of Periodontal Research*.

[9] Bosshardt D. D. (2005). Are cementoblasts a subpopulation of osteoblasts or a unique phenotype?. *Journal of Dental Research*.

[10] Tang Y., Wu X., Lei W. (2009). TGF-beta1-induced migration of bone mesenchymal stem cells couples bone resorption with formation. *Nature Medicine*.

[11] Bismar H., Klöppinger T., Schuster E. M. (1999). Transforming growth factor *β* (TGF-*β*) levels in the conditioned media of human bone cells: relationship to donor age, bone volume, and concentration of TGF-*β* in human bone matrix in vivo. *Bone*.

[12] Janssens K., ten Dijke P., Janssens S., Van Hul W. (2005). Transforming growth factor-*β*1 to the bone. *Endocrine Reviews*.

[13] Liu J., Zhao Z. H., Li J. (2009). Hydrostatic pressures promote initial osteodifferentiation with ERK1/2 not p38 MAPK signaling involved. *Journal of Cellular Biochemistry*.

[14] McCauley L. K., Nohutcu R. M. (2002). Mediators of periodontal osseous destruction and remodeling: principles and implications for diagnosis and therapy. *Journal of Periodontology*.

[15] Khosla S. (2001). Minireview: the OPG/RANKL/RANK system. *Endocrinology*.

[16] Boabaid F., Berry J. E., Koh A. J., Somerman M. J., McCauley L. K. (2004). The role of parathyroid hormone-related protein in the regulation of osteoclastogenesis by cementoblasts. *Journal of Periodontology*.

[17] Quinn J. M. W., Itoh K., Udagawa N. (2001). Transforming growth factor *β* affects osteoclast differentiation via direct and indirect actions. *Journal of Bone and Mineral Research*.

[18] Dalla-Bona D. A., Tanaka E., Inubushi T. (2008). Cementoblast response to low- and high-intensity ultrasound. *Archives of Oral Biology*.

[19] Dereka X. E., Markopoulou C. E., Vrotsos I. A. (2006). Role of growth factors on periodontal repair. *Growth Factors*.

[20] Fox S. W., Lovibond A. C. (2005). Current insights into the role of transforming growth factor-*β* in bone resorption. *Molecular and Cellular Endocrinology*.

[21] Pavlin D., Gluhak-Heinrich J. (2001). Effect of mechanical loading on periodontal cells. *Critical Reviews in Oral Biology and Medicine*.

[22] Nagatomi J., Arulanandam B. P., Metzger D. W., Meunier A., Bizios R. (2003). Cyclic pressure affects osteoblast functions pertinent to osteogenesis. *Annals of Biomedical Engineering*.

[23] Liu J., Zhao Z., Zou L. (2009). Pressure-loaded MSCs during early osteodifferentiation promote osteoclastogenesis by increase of RANKL/OPG ratio. *Annals of Biomedical Engineering*.

[24] Shen J., Li S., Chen D. (2014). TGF-*β* signaling and the development of osteoarthritis. *Bone Research*.

[25] Kuchler U., Schwarze U. Y., Dobsak T. (2014). Dental and periodontal phenotype in sclerostin knockout mice. *International Journal of Oral Science*.

[26] Furfaro F., Ang E. S. M., Lareu R. R., Murray K., Goonewardene M. (2014). A histological and micro-CT investigation in to the effect of NGF and EGF on the periodontal, alveolar bone, root and pulpal healing of replanted molars in a rat model—a pilot study. *Progress in Orthodontics*.

[27] Kagayama M., Akita H., Sasano Y., Kindaichi K. (1994). Localization of uncalcified cementum in adult rat molar roots and its relation to physiological tooth movement. *Archives of Oral Biology*.

